# Chronic Traumatic Sagittal Band Injury with Extensor Tendon Dislocation: Report of a Case and New Surgical Technique

**DOI:** 10.5704/MOJ.1707.007

**Published:** 2017-07

**Authors:** SY Or, YC Khaw, PX Hwang, TK Ong

**Affiliations:** Department of Orthopaedics, Seberang Jaya Hospital, Prai, Malaysia

**Keywords:** extensor tendon dislocation, sagittal band injury, surgical technique

## Abstract

Chronic sagittal band injury with tendon dislocation of the extensor digitorum communis in the hand often requires operative stabilization. Various surgical techniques have been reported to repair and reconstruct the sagittal band. Nonetheless, most of the techniques are technically demanding and require donor graft. In this case report, we report a novel surgical technique to centralize and stabilize the tendon by reattaching the radial sagittal band with anchor sutures. The advantages of this new technique are simple, no donor morbidity and stable repair to restore the normal biomechanics of the tendon. The patient was able to return to work in three months and no recurrent dislocation was noted at review two years after surgery.

## Introduction

Sagittal band injury with dislocation of the extensor digitorum communis tendon (EDC) is rare. It is frequently associated with blunt trauma. Middle finger is the most commonly affected^1^. The clinical presentation includes pain, swelling and snapping over the affected knuckle associated with limitation in finger flexion. Most of the reconstructive surgeries reported in literature require tendon graft from lumbricals, juncturae tendinum, extensor tendon or palmaris longus. We describe a new surgical technique in this case, whereby the radial sagittal band was anchored into the metacarpal head without donor tendon.

## Case Report

**Fig. 1:**
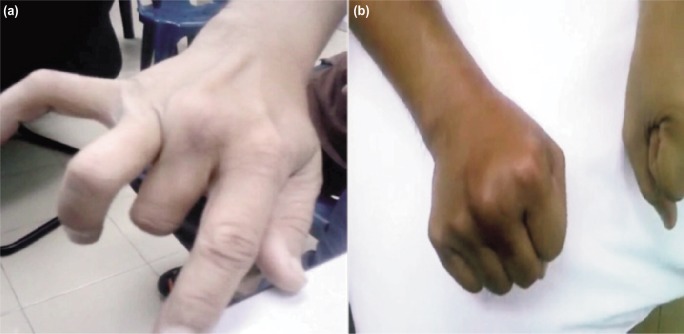
(a) Dislocation of the EDC and ROM pre-operative and (b) Stability of EDC and ROM of MCPJ post-operative.

A 34 year-old right-handed male, armed security guard, presented with painful clicking over the right third knuckle for four months after a fall while riding motorcycle. This injury had prevented him from performing firearm shooting practice. On examination, the EDC tendon of the middle finger was found to be dislocating ulnarwards during active metacarpal-phalangeal joint (MCPJ) flexion and reducing during extension of the joint, causing intense pain at the joint. The finger range of motion (ROM) was minimally reduced (Fig. 1a) but the grip strength was markedly reduced compared to the uninjured side. Dynamic ultrasound and magnetic resonance imaging (MRI) study of the right middle finger confirmed the diagnosis. The patient was counselled for surgery in view of chronic symptomatic injury and unsuccessful splinting.

Under general anaesthesia, a four-centimetre longitudinal dorsal skin incision was made over the 3rd MCPJ with a gentle curve to avoid metacarpal head prominence. The entire extensor mechanism over the MCPJ was then exposed. The contracted scared ulnar sagittal band was released. Juncturae tendinum between third and fourth EDC tendon was released from the fourth extensor tendon. The radial sagittal band was then released from scar tissue to improve the long tendon gliding. Post scar debridement, full ROM was achieved over the MCPJ, proximal interphalangeal joint (PIPJ) and distal interphalangeal joint (DIPJ).

**Fig. 2:**
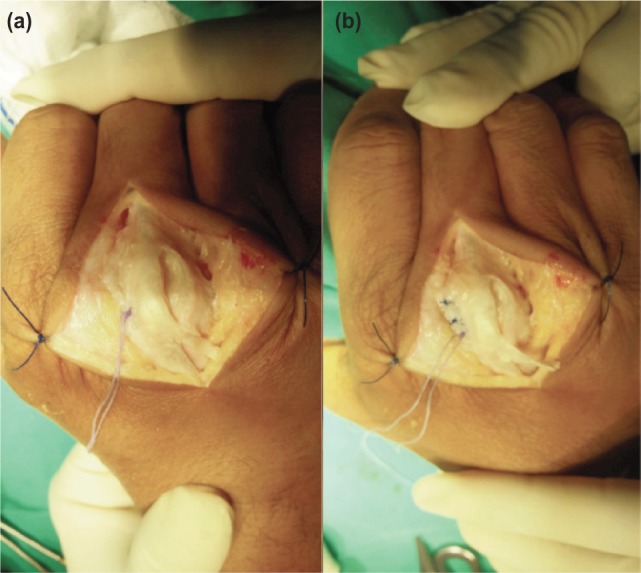
(a) Ulnar juncturae tendinum, ulnar sagittal band and torn scared radial sagittal band released. Anchor suture implanted to metacarpal bone radial-dorsally and anchor suture applied in tension-free fashion with MCPJ and PIPJ in full flexion and (b) Completion of radial sagittal repair distally. Note the position of anchor suture being proximal to the rest of the repair.

An anchor suture [DePuy Mitek micro anchor 3/0] was inserted at the junction between the head and neck of the 3rd metacarpus from radial-dorsally towards ulnar-palmar. The radial sagittal band was then tied to the metacarpal bone with sutures as illustrated (Fig. 2a). All of MCPJ, PIPJ and DIPJ were flexed in full during repair to prevent loss of flexion range post-operatively. After excision of scar, the remaining distal part of the radial sagittal band was repaired directly to the remnants of volar sagittal band using absorbable suture as illustrated (Fig. 2b) with the MCPJ, PIPJ and DIPJ completely flexed. The stability of the EDC tendon was assessed throughout flexion and extension. The surgical wound was closed with subcuticular absorbable sutures. The upper limb was immobilized with a below elbow volar splint with MCPJ flexion at 60 degree, wrist in 30 degree extension and interphalangeal joint in extension.

A thermoplastic splint was kept for six weeks. Passive and active ROM exercises were initiated with gradual increment in range over six weeks. The splint was removed on the 6th week, followed by gradual strengthening exercise. Three months later, range of movements over the third MCPJ and all the other finger joints were full (Fig. 1b). The grip strength had improved two-fold to 28kg. Optimum fine motor function was also achieved. He resumed working three month after operation and was able to perform firearm shooting at six months. The grip strength of the finger was regained to normal at one year and maintained at review at two years (Table I). No recurrent dislocation or other complication were noted.

**Table I:** Comparing pre-operative and post-operative functional

**Outcome parameter**	**Before operation**	**3 months post-operation**	**2 years post-operation**
Right MCPJ active ROM	0-80 degrees	Full	Full
EDC ‘snapping’	Yes	No	No
Grip Strength of affected limb (Right)	3-11kg	28kg	40 kg
Grip strength of unaffected limb (Left)	41-42kg	39kg	42 kg
Hand Function (fine motor)	Impaired	Comparable to contralateral side	Comparable to contralateral side
Pain Score/VAS	3	0	0

## Discussion

A new surgical method was devised based on the classic technique of realignment of EDC tendon and repair of the sagittal band defect. Centralization of ulnarly dislocated EDC tendon was achieved by releasing the contracted ulnar sagittal band and then the ulnar juncturae tendinum. Complete release of the ulnar sagittal band will not cause radial subluxation of the tendon^1^. The extensor tendon was stabilized by anchoring the proximal radial sagittal band to the metacarpus at an isometric point. The anchor suture also prevented the EDC from bowstringing and ensured EDC remained over the dorsal midline of MCPJ throughout all ranges of motion at wrist, MCPJ, PIPJ and DIPJ.

**Fig. 3:**
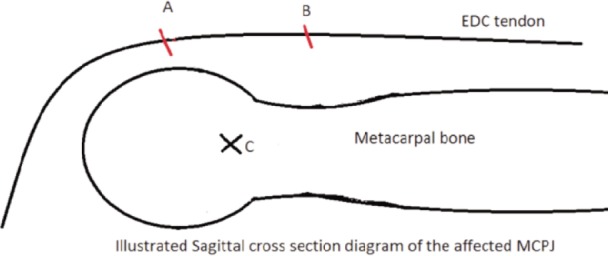
This diagram demonstrates sagittal cross section of metacarpal bone with the extensor digitorum communis tendon. Point A is the referral point when MCPJ and PIPJ in 90 degree flexion while point B is reflection of point A when both the MCPJ and PIPJ in extension. Point C (isometric point) is the proposed anchor suture position. The anchor suture should be placed so that point AC and BC are equal. Point A should be at the proximal part of radial sagittal band which have greater contribution to extensor tendon stability.

There are two critical steps in this repair. Firstly, the anchor suture was inserted at the junction between metacarpal head and neck radially, creating a volar-radial vector for the radial sagittal band repair (Fig. 3). Too distal placement of the anchor suture should be avoided as it may impede MCPJ flexion^2^. In a biomechanical study, Rayan and Young^1^concluded that partial sectioning of the proximal part of the radial sagittal band is sufficient to produce ulnar subluxation of the extensor tendon. Similar finding was not observed in partial sectioning of the distal part of the band. Thus, the anchor suture is placed at the metacarpal neck to repair the proximal part of the radial sagittal band. The distal part was then repaired with a few interrupted sutures.

Secondly, all small joints of the hand were kept in full flexion during knot tying to secure the sagittal band down to the metacarpus. In addition, the wrist was kept in neutral position. In the similar study by Young and Rayan^1^, the average pressure generated inside the sagittal band was the highest when the MCP joint was fully flexed. Thus if repair is done in a position other than full flexion, excessive high pressure over the repaired sagittal band may result in early failure.

This reconstruction surgery is recommended for chronic injury as acute injury can be treated conservatively with splint if presented less than 2-4 weeks of injury^2^. Patients who fail splinting and have persistent pain are candidates for reconstructive surgery. Contraindications for reconstructive surgery include joint contracture, arthritis deformity and degenerative age-related laxity of tendons.

Various surgical techniques have been described in the literature to achieve stability of the EDC tendon, ranging from direct repair to soft tissue reconstruction of the sagittal band. Direct repair of the sagittal band may not be possible in chronic injury due to contracted remnant of the band within the scar tissue. Numerous types of soft tissue reconstructions of the radial sagittal had been reported to solve this problem. These techniques vary in donor graft and its path pattern. Commonly harvested autografts include lumbricals, slip of extensor tendon^3^, palmaris longus^2^ and ulnar juncturae tendinum^4^. These grafts were then rerouted and sutured to joint capsule, bone tunnel^2^, radial collateral ligament^3^, deep transverse metacarpal ligament^4^ or looping over the lumbrical.

Watson *et al*^5^ described a reconstruction method which entails weaving a retrograde segment of extensor tendon upon itself after passing it through the deep transverse metacarpal ligament. The deep transverse metacarpal ligament was used as a pulley to maintain the centralized position of extensor tendon. Watson’s method requires accurate repositioning of extensor tendon, proper placement of sagittal band with relation to the metaphalangeal joint and a repair of sufficient strength for a successful outcome^5^.

Reconstruction using soft tissue is technically more demanding and requires considerable skill in harvesting a graft successfully. With anchor sutures, tedious graft harvesting and potential morbidity at donor site can be prevented. This new surgical technique is less time consuming as graft harvesting is not required and only a single skin incision is made. Compared to other methods, this new surgical technique is relatively simple and stable for faster recovery. In short, this simple new devised technique shortens the surgical time, reduces patient morbidity and speeds up patient recovery time.
